# Evidence for Seed Transmission of *Xylella fastidiosa* in Pecan (*Carya illinoinensis*)

**DOI:** 10.3389/fpls.2022.780335

**Published:** 2022-04-08

**Authors:** Kimberly Cervantes, Angelyn E. Hilton, Rio A. Stamler, Richard J. Heerema, Clive Bock, Xinwang Wang, Young-Ki Jo, L. J. Grauke, Jennifer J. Randall

**Affiliations:** ^1^Molecular Biology and Interdisciplinary Life Sciences, New Mexico State University, Las Cruces, NM, United States; ^2^United States Department of Agriculture, Southern Plains Agricultural Research Center, Pecan Breeding and Genetics, Somerville, TX, United States; ^3^Entomology, Plant Pathology, and Weed Science, New Mexico State University, Las Cruces, NM, United States; ^4^Extension Plant Sciences, New Mexico State University, Las Cruces, NM, United States; ^5^United States Department of Agriculture, Southeastern Fruit and Tree Nut Research Laboratory, Byron, GA, United States; ^6^Department of Plant Pathology and Microbiology, Texas A&M University, College Station, TX, United States

**Keywords:** pecan bacterial leaf scorch, phytopathology, bacteriology, epidemiology, seedling development

## Abstract

Pecan bacterial leaf scorch, caused by *Xylella fastidiosa* subsp. *multiplex*, is an economically significant disease of pecan with known detrimental effects on the yield of susceptible cultivars. In this study, endosperm was harvested from developing pecan seeds, and direct qPCR and sequencing were used to detect and confirm the presence of *X. fastidiosa*. DNA was isolated from mature seeds originating from seven trees, revealing a positivity rate up to 90%, and transmission of *X. fastidiosa* from infected seed to the germinated seedlings was found to be over 80%. Further epidemiological analyses were performed to determine where *X. fastidiosa* localizes in mature seed and seedlings. The highest concentrations of *X. fastidiosa* DNA were found in the hilum and outer integument of the seeds and the petioles, respectively. High-, medium-, and low-density seeds were harvested to determine the impact of the bacterium on seed density and seedling growth rate. The growth rate of seedlings originating from low-density seeds was significantly reduced compared to the medium- and high-density seeds. Despite the increased growth and germination rates, the high-density seed group had a greater proportion of samples that tested positive for the presence of *X. fastidiosa* by qPCR. The results demonstrate the ability of *X. fastidiosa* to colonize developing seeds and be efficiently transmitted from well-developed seeds to germinated seedlings. Continued research is needed to understand the plant-microbe interactions involved in the colonization of pecan seeds by *X. fastidiosa* and to develop effective phytosanitary approaches to reduce the risks posed by seed transmission.

## Introduction

Pecan bacterial leaf scorch (PBLS), caused by *Xylella fastidiosa* subsp. *multiplex* (family Xanthomonadaceae), is a chronic disease that is widespread across the southern pecan growing region of the United States (Sanderlin and Heyderich-Alger, 2000; Vitanza, 2015; Hilton et al., 2017; Bock et al., 2018). PBLS symptoms include tan to brown leaf tips and/or edges, uniform necrosis along leaf margins toward the midrib of leaflets, and leaf abscission (Sanderlin and Heyderich-Alger, 2000). The pathogen *X. fastidiosa* is a xylem-limited, gram-negative bacteria known to cause disease in a broad range of host plants, including grape, oak, peach, almond, plum, sycamore, olive, citrus, and other hardwood trees (EFSA, 2020). Disease symptoms caused by *X. fastidiosa* are thought to be due to the accumulation of the bacteria in the xylem, depriving the plant of water and nutrients, resulting in symptoms similar to those of drought, nutrient deficiency, and salt damage (Newman et al., 2003; Chatterjee et al., 2008; Oliver et al., 2015). In addition, recent research in grapevines identified the host stress response to the modification of xylem microenvironments by *X. fastidiosa*, through the secretion of enzymes, biofilms, and other compounds (Nascimento et al., 2016; Dandekar et al., 2019). Severe PBLS disease symptoms have been reported to result in up to 58% premature defoliation by the end of the growing season in the susceptible cultivar (cv.) Cape Fear (Sanderlin and Heyderich-Alger, 2003). Expression of PBLS symptoms can have other detrimental effects on pecan morphology and seed quality, including reduced terminal bud weight and seed density, respectively. In cv. Cape Fear, a 10–13% reduction of in-shell seed weight and a 14–19% reduction in kernel weight, was found in symptomatic trees compared to asymptomatic trees (Sanderlin and Heyderich-Alger, 2003). These losses lead to a decrease in total product yield, posing an economic threat to growers and the pecan industry.

*X. fastidiosa* colonizes the tracheids and vessel elements of its plant hosts, with movement both vertically and laterally through the breach of pit membranes (Mollenhauer and Hopkins, 1974; Roper et al., 2007; Ellis et al., 2010; Cardinale et al., 2018). *X. fastidiosa* is transmitted in pecan by insect vectors and through the grafting of scions to rootstock (Sanderlin, 2005; Sanderlin and Melanson, 2006, 2010). The vectors of the pathogen are all xylem-feeding suctorial insects (Purcell and Finlay, 1979). In a study by Sanderlin and Melanson (2010), species of the Cicadellidae (leafhoppers) and Cercopidae (spittlebugs) families were assessed to determine the transmission rates of *X. fastidiosa* subsp. *multiplex* to pecan seedlings. Sharpshooters (*Homalodisca* spp.) had transmission rates of *X. fastidiosa* of approximately 10–20%, while the pecan spittlebug (*Clastoptera achatina*) was able to cause infection at a rate of 11%. Graft transmission is also a potential source of infection since improved pecan cultivars are clonally propagated onto seedling-derived rootstock. Sanderlin (2005) successfully grafted 12 uninfected scions onto PBLS-infected rootstocks and found that 10 of the 12 (>85%) developed leaf scorch symptoms and tested positive for *X. fastidiosa* by enzyme-linked immunosorbent assay (ELISA) over a two-year period. Likewise, 105 *X. fastidiosa-*infected scions were grafted onto non-infected rootstocks revealing a transmission rate of 21.3% (Sanderlin and Melanson, 2008).

Evidence for alternative modes of transmission of *X. fastidiosa* has been limited in pecan and other plant hosts. The probability of seed-to-seedling transmission was proposed in citrus variegated chlorosis (CVC) of sweet orange (*Citrus* x *sinensis*), where *X. fastidiosa* subsp. *pauca* has been detected in fruit, seed coats, and embryos of three cultivars (Li et al., 2003; Coletta-Filho et al., 2014; Hartung et al., 2014). Isolates were obtained from symptomatic citrus seedlings germinated from putative *Xylella-*infected seed (Li et al., 2003), but follow-up attempts failed to isolate or detect the bacterium in seedlings (Coletta-Filho et al., 2014; Hartung et al., 2014). A recent study reported the presence of *X. fastidiosa* in the endosperm of undeveloped pecan seeds, and so the suggestion that *X. fastidiosa* can reside in pecan seed is not unprecedented and merits further investigation (Hilton et al., 2020). It is unknown if the bacterium also colonizes mature seed and can be transmitted from seed to germinated seedling. Propagation of improved pecan cultivars occurs by grafting clonal scions onto genetically diverse rootstock seedlings (Worley, 1994). The rootstock is cultivated from seedlings germinated from open-pollinated seeds prior to grafting. The presence of *X. fastidiosa* in seeds may have consequential impacts on the horticultural practices of the industry, particularly given the high rates of transmission from grafting uninfected scions onto *Xylella-*infected rootstocks (Sanderlin, 2005). High-density seeds are recognized as optimal for germination, and since disease symptoms caused by PBLS can negatively affect in-shell seed and kernel weight, it is critical to determine what influences the presence of the bacterium within seed may have on the overall quality (Grauke and Thompson, 1995; Sanderlin and Heyderich-Alger, 2003).

Pecan is one of the 20 recognized species of hickory, which are native to North America, and is a highly valued, cultivated nut crop in the US with an estimated value of $469 million per year (Rüter et al., 1999; Marzolo, 2015; USDA N.A.S.S., 2020). Pecan production has expanded to South Africa, Australia, China, Uruguay, Argentina, and Brazil, and cultivation of pecan is expected to increase over the next 30 years (Wood et al., 1990; Wakeling et al., 2001; Lazarotto et al., 2014; Zhang et al., 2015). Seed transmission increases the likelihood of *X. fastidiosa* dissemination to pecan-producing regions previously free of the pathogen, including those outside the native range. Such is the case for Olive Quick Decline Syndrome (OQDS), where the recent introduction of *X. fastidiosa* subsp. *pauca* to south-eastern Italy has led to severe infection and the death of over a million olive trees (Martelli et al., 2016; Morelli et al., 2021). International distribution of pecan budwood or scion material from the USDA-ARS National Collection of Genetic Resources for Pecans and Hickories (NCGR-*Carya*) was halted in 2015 due to the endemic nature of *X. fastidiosa* (Grauke et al., 2016; Hilton et al., 2017). Heat treatment of graftwood has been developed but is not completely effective in eliminating *X. fastidiosa*, and no treatment is currently available for seed or rootstock material (Sanderlin and Melanson, 2008; Hilton et al., 2021). Phytosanitary distribution requirements have not yet been evaluated for pecan seeds. More complete knowledge of the modes of transmission for *X. fastidiosa* will allow researchers and commercial pecan growers to develop new treatments and to apply integrated pest management (IPM) practices for disease mitigation. The purpose of this study is to evaluate the transmission of *X. fastidiosa* from pecan seed to germinated seedlings, including rates of transmission, localization patterns of the pathogen, and the overall impact on seed density.

## Materials and Methods

### Genomic DNA Extraction

Total gDNA was extracted from pecan tissues using either modified CTAB methods derived from Grauke et al. (2011) and Paterson et al. (1993), or the Qiagen DNeasy Plant Mini kit (#69106, Qiagen, Hilden, Germany). CTAB methods included the brief grinding of frozen tissue in liquid nitrogen and treatment with an extraction buffer [0.1 M Tris–HCl pH = 8, 1.4 M NaCl, 0.02 M Na2EDTA pH = 8, 2% (w/v) CTAB, 0.2% (v/v) of β-mercaptoethanol, and 2% (w/v) polyvinylpyrrolidone (PVPP)] on a 65°C hotplate for 30 min. gDNA was extracted with chloroform/isoamyl alcohol (24:1) and phenol/chloroform/isoamyl alcohol (25:24:1) and precipitated with isopropanol and ethanol. gDNA samples isolated using the CTAB method were purified using the Genomic DNA Clean and Concentrator kit (#D4067, Zymo Research, Irvine, CA) to remove contaminants that might inhibit downstream analysis. Total gDNA extracted *via* the Qiagen DNeasy Plant Mini Kit (#69106, Qiagen, Hilden, Germany) was based on the manufacturer’s protocol. DNA concentration and quality were measured using a NanoDrop 2000 ultraviolet (UV)-Vis spectrophotometer (ThermoFisher Scientific, Waltham, MA) or an IMPLEN NanoPhotometer P-Class 360 (Westlake Village, California). All DNA samples were stored in TE or nuclease-free dH_2_O at-20°C prior to the downstream application to prevent DNA degradation.

### Traditional PCR and Real-Time Quantitative PCR Analysis

Previously published primers for the detection of *X. fastidiosa* were used for PCR analysis: the HL-5 and HL-6 (Francis et al., 2006), and NMU3 forward and reverse (Hilton et al., 2020) primers. PCR reactions were conducted on a BioRad CFX96 real-time Thermal Cycler using either iTaq SYBR Green polymerase (#1725120, BioRad, CA) or SsO Advanced SYBR Green polymerase (#1725270, BioRad). Reactions consisted of 2X polymerase master mix, respective to each kit, 0.2 μM forward primer, 0.2 μM reverse primer, DNA template, and molecular grade water to a volume of 20 μL. Two-step PCR was utilized for all reactions; denaturation was performed at 95°C followed by the annealing and extension temperature at 62°C for 39 cycles, as previously described (Hilton et al., 2020). A positive control [either purified *X. fastidiosa* DNA or lyophilized *X. fastidiosa* supplied by Agdia Inc. (#LPC 34503, Agdia Inc., Elkhart, IN)] and a non-template control (nuclease-free dH_2_O) were included. The amplified products were resolved on either a 1.5% or 2% agarose gel stained with ethidium bromide and visualized under UV light.

The previously reported TaqMan probes and primer sets specific to the uncharacterized HL protein of *X. fastidiosa* subsp. *multiplex* and the actin-encoding gene of *Carya illinoinensi*s were used for qPCR amplification (Randall et al., 2015; Hilton et al., 2020). The amplification of the actin loci of *C. illinoinensis* served as the internal positive control (IPC) to confirm absence of inhibitors and to normalize the estimated *X. fastidiosa* DNA concentration. All qPCR reactions were prepared using optical 96-well plates and the TaqPath ProAmp Master Mix (#A30871, ThermoFisher Scientific, Waltham, MA) as the reagent chemistry. The qPCR profile using standard cycling was performed based on the manufacturer’s protocols specific for plant genotyping. Fluorescent intensities in each reaction well were determined by an Applied Biosystems StepOne Real-Time PCR system (ThermoFisher Scientific). A final gDNA concentration of 1 ng/μl was used as the template for each unknown sample reaction, as well as six negative, non-template controls consisting of dH₂O and qPCR reagents. A positive control consisting of purified *X. fastidiosa* gDNA was serial diluted [1:10 (1, 0.1, 0.01, 0.001, and 0.0001 ng per 10 μl reaction)] to create a standard curve. The Ct values corresponding to the successful amplification of *X. fastidiosa* DNA were normalized to the Ct values of actin from each sample using the 2^-ΔΔCT^ method (Schmittgen and Livak, 2008). For each experiment, the normalized Ct values from the pecan density seed amplification was used as the control for comparison.

### Sequencing

DNA amplicon bands corresponding to the correct size were either excised from gels and purified using a Qiaquick Gel Extraction kit (#28704, Qiagen) or treated with ExoSAP IT (#78250.40UL, ThermoFisher Scientific). Sequencing reactions were performed with either a BigDye Terminator v3.1 Cycle Sequencing kit (#4337458, ThermoFisher Scientific), according to the manufacturer’s instructions, and separated on an ABI 3130xL Genetic Analyzer at NMSU Molecular Biology facility (ThermoFisher Scientific); or sent to the Molecular Cloning Laboratories (San Francisco, CA) for sequencing. Amplicons were sequenced in both the forward and reverse directions and were paired in a consensus sequence before sequence alignment analysis. There were approximately 108 amplicons sequenced for this study. Sequence chromatograms were evaluated for sequencing quality, and alignments were performed using Geneious Prime (Geneious version 2021.1 created by Biomatters).^1^ Sequences were trimmed at the terminal ends to remove primer sequences and/or low-quality reads, which reduced the size of the amplicon. Representative HL sequences from various strains of *X. fastidiosa* were obtained from GenBank (Bethesda, MD)^2^ and used for sequence alignment comparisons using Geneious Prime (Kearse et al., 2012) with free end gaps and a 65% cost matrix. A phylogenetic tree was constructed using the HL amplicon sequences and *X. fastidiosa* HL sequences acquired from GenBank using the Nearest Neighbor method (HKY) with XF 9a5c (gb | AE003849.1) as the outgroup using Geneious Prime 2. The tree was bootstrapped 1,000 times had 70% support threshold.

### Liquid Endosperm

One hundred and eighty seeds at water stage were collected from 18 pecan trees (10 seeds each from cvs. Bradley, Wichita, Mandan, Lakota, and Nacono mother trees) in Bowie, AZ (Lat. 32°19′29.01″N, Lon. 109°29′08.94″W, El. 1148.18 m) in July 2017. The pecan seed shuck (involucre) and shell tissues were removed to expose the integument to extract the liquid endosperm without contaminating it with infected maternal tissue. Once the integument containing the liquid endosperm was exposed, a sterile syringe was injected into the integument, and the liquid endosperm was extracted. The liquid endosperm from each sample was diluted 1:10, 1:50, 1:75, 1:100, and 1:1000 (v/v) in molecular grade dH_2_O in case of PCR inhibition, and the presence of *X. fastidiosa* was detected by direct qPCR using the HL-5/HL-6 and NMU3 primer sets, as previously described (Hilton et al., 2020).

### Seed Collection and DNA Isolation From Seeds

Fully developed pecan seeds (*N* = 336) from six trees (Table 1) located throughout Las Cruces, NM and one tree [cv. Cape Fear, CSV 18–11 (TX1)] from the USDA-ARS NCGR-*Carya* in Somerville, TX, were randomly collected in November and December of 2015. The cultivars and ages of the selected non-commercial pecan trees (designated NMU1-NMU6) from which the seeds were sampled were unknown. However, these trees were known to have PBLS, or to have displayed symptoms of PBLS and thus were suspected of being infected with *X. fastidiosa*. Total gDNA was extracted from the kernels of 10 seeds from TX1-NMU5 and 12 seeds from NMU6 (72 seeds total) using a Qiagen DNeasy Plant Mini Kit, as previously described. The remaining seeds (114 seeds) from NMU1-NMU5 and 20 seeds from TX1 were stratified and planted in Lambert’s (Quebec, Canada) potting soil and grown as seedlings in an insect-free quarantine greenhouse at a temperature of 29–35°C for approximately 3 months (Table 1). All seedlings were dissected, and the leaves, stems, tap roots, and fibrous roots were stored individually in a −20°C freezer until DNA extraction. Total gDNA of seedling tissues was isolated using a DNeasy Plant Mini Kit (Qiagen), followed by qPCR analysis using the HL5/HL6 primer sets, as previously described, to determine the presence of *X. fastidiosa* DNA within each subsample. Sequencing of the HL amplicon from a subset of samples was performed, as previously described, to ensure that amplification was of the correct locus.

**Table 1:** Seed collection, germination, and diagnoses.

Tree sample name	Latitude and longitude	Cultivar	Number of seeds collected	Purpose	Number of seeds tested	% of seeds positive for *X. fastidiosa*	Number of seeds that germinated	Number of seedlings that tested positive for *X. fastidiosa*
TX1	30°31′17.10′′N, 96°25′36.68′′W	Cape Fear (CSV 18–11)	30	DNA Extraction Propagation	10	80	0 out of 20	N/A
TX1	30°31′17.10′′N, 96°25′36.68′′W	Cape Fear (CSV 18–11)	150	Seed Density	N/A	N/A	51 out of 150	N/A
NMU 1	32°16′59.48′′N, 106°44′57.69′′W	Unknown	30	DNA Extraction Propagation	10	50	11 out of 20	8 out of 11
NMU 2	32°16′59.48′′N, 106°44′57.69′′W	Unknown	30	DNA Extraction Propagation	10	70	6 out of 20	6 out of 6
NMU 3	32°16′41.30′′N, 106°46′25.71′′W	Unknown	30	DNA Extraction Propagation	10	67	11 out of 20	11 out of 11
NMU 4	32°16′41.30′′N, 106°46′25.71′′W	Unknown	30	DNA Extraction Propagation	10	90	2 out of 20	N/A
NMU 5	32°16′41.30′′N, 106°46′25.71′′W	Unknown	24	DNA Extraction Propagation	10	50	2 out of 14	2 out of 2
NMU	6 32°16′43.38′′N, 106°45′25.54′′W	Unknown	12	DNA Extraction	12	83	N/A	N/A

Indicates the geolocation for seed collection, the percentage of seeds that tested positive for *X. fastidiosa*, number of seeds that germinated, and the resulting number of seedlings that tested positive for *X. fastidiosa*.

### Pecan Seed Measurements and Germination

A total of 150 mature seeds were harvested from an *X. fastidiosa*-positive tree, TX1 from the USDA-ARS NCGR-*Carya* “CSV” orchard in Somerville, TX (Table 1). The seeds were separated into three categories based on seed density. Seed density (g/cm^3^) was measured by determining the dry weight (g) of each seed divided by the volume (cm^3^), estimated as a function of buoyancy force. The classification of seed density based on the average of each group was designated as follows: high seed density (0.72 g/cm^3^), medium seed density (0.62 g/cm^3^), and low seed density (0.52 g/cm^3^). Fifty seeds were tested per seed density group. The sampled seeds were bisected into halves, and the half containing the embryonic shoot-root axis was planted into a sterilized, germinating soil mixture. The seeds were germinated in an insect-free, quarantined plant growth chamber at 23°C with a 16-h photoperiod. The developing seedlings were monitored for 46 days for growth rate, percent germination, and the average days to germination within each seed density group. Plant height, as a function of growth rate, was measured every 3 days from the soil surface to the shoot apical meristem with a metric ruler. An analysis of variance (ANOVA) or an analysis of covariance (ANCOVA) was performed for grouped comparisons, followed by a *post hoc* Tukey’s HSD test for mean separation (*α* = 0.05) using SAS University Edition (SAS, Cary, NC) to determine any differences in the days to germination and growth rate, respectively, of seedlings originating from the low-, medium-, and high-density seed groups. A *χ*^2^ analysis was performed (SAS University Edition) to determine whether there were significant differences (*p* < 0.05) in the proportion of seeds that germinated out of the 50 seeds planted from each seed density group.

The remaining seed halves (not containing the embryo) of the cv. Cape Fear (TX1) seeds were frozen and ground in liquid nitrogen prior to total gDNA isolation. gDNA isolation of reserved seed halves was performed using the modified CTAB method (Grauke et al., 2011), followed by purification with the Zymo Genomic DNA Clean and Concentrator kit (Zymo Research), as previously described. qPCR analysis was performed, as previously described, to determine the presence of *X. fastidiosa* DNA within the different seed density groups. A *χ*^2^ analysis was performed (SAS University Edition) to determine significant differences (*p* < 0.05) in the proportion of seeds that tested positive for the presence of *X. fastidiosa* per the total number of seeds in each group.

### Pecan Tissue Dissections for *X. fastidiosa* DNA Quantification

The presence and concentration of *X. fastidiosa* were compared in different anatomical components of TX1 seeds. Four seeds were randomly selected from the mid-canopy from the TX1 tree. Following harvest, 10 mg subsamples from the (a) embryo, (b) hilum, (c) vascular integument, and (d) outer integument were dissected from the kernel using a sterile scalpel and immediately frozen in liquid nitrogen. The vascular and outer integument could not be isolated independently, so samples included both the seed coat and cotyledon tissue.

To determine where *X. fastidiosa* is localized within germinated seedlings, eight open-pollinated TX1 seedlings from the previous seed density assay were selected based on the expression of symptoms. The seedlings all originated from the high-density seed group and were propagated as previously described. The seedlings were harvested after 3 months of growth and dissected into four anatomical parts, (a) petioles, (b) shoots, (c) leaflets, and (d) roots. A 10 mg sample of each bulked plant part was frozen and ground in liquid nitrogen prior to total gDNA isolation.

For both experiments, total gDNA was isolated using the DNeasy Plant Mini kit (Qiagen), followed by qPCR analysis to determine the presence and concentration of *X. fastidiosa* DNA within each subsample, as previously described. Special attention was taken when performing gDNA isolation of seed structures to ensure the limited carryover of polysaccharides, a possible qPCR inhibitor (Schrader et al., 2012). Significant differences between grouped comparisons of *X. fastidiosa* DNA concentration represented by normalized 2^-ΔΔCt^ within each anatomical part of seeds and seedlings, respectively, were determined by ANOVA, followed by a post-hoc Tukey’s HSD mean separation (SAS University Edition).

## Results

### Evidence of *X. fastidiosa* in Developing and Mature Pecan Seeds

**Figure 1:**
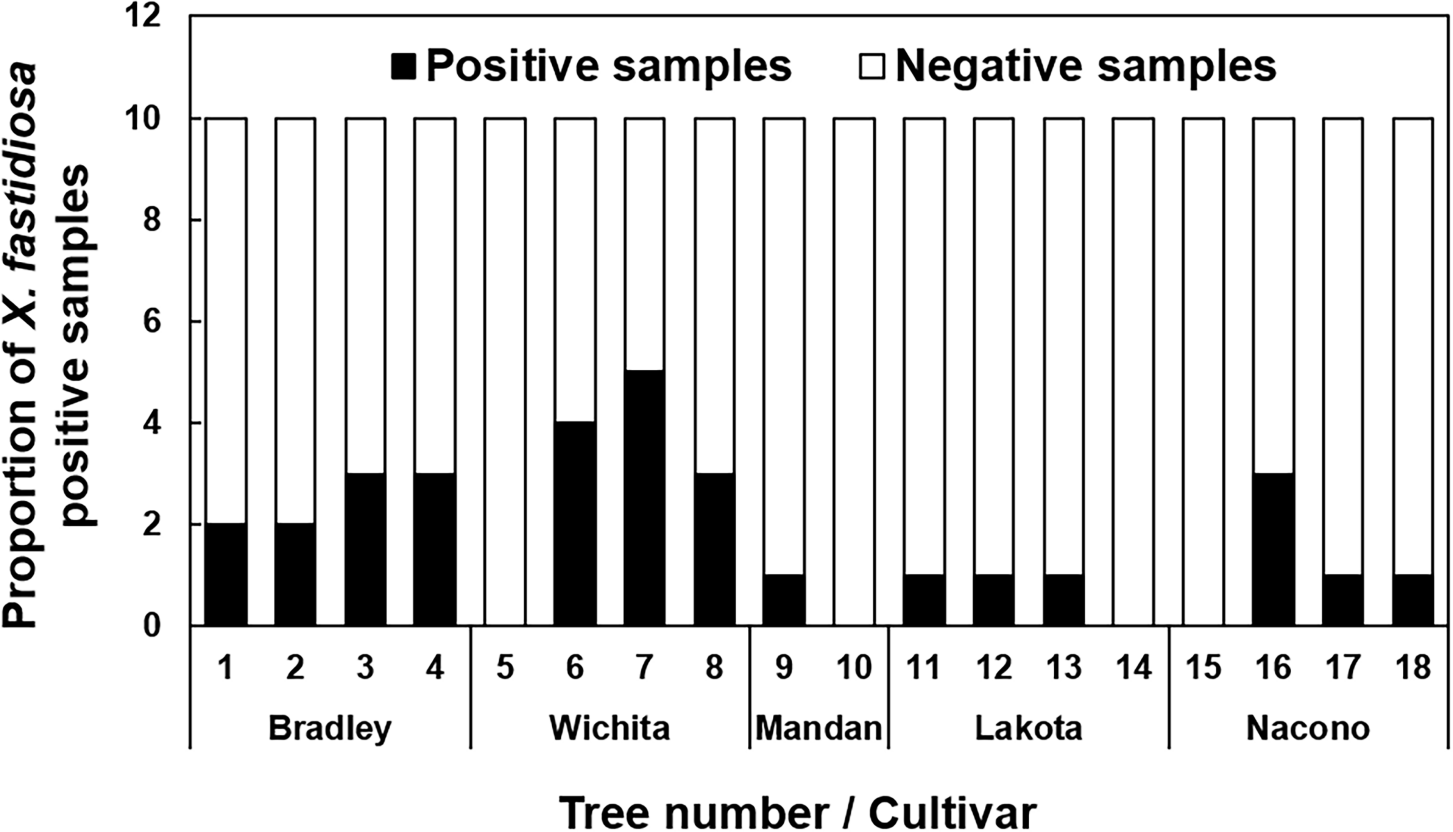
Incidence of pecan liquid endosperm positive for *Xylella fastidiosa*. Indicates the proportion of seed endosperm that tested positive for the presence of *X. fastidiosa*. The liquid endosperm of collected pecan seeds from NM and AZ pecan trees were analyzed for the presence of *X. fastidiosa* using qPCR. A total of 180 seeds were tested. Black bars indicate the total number of samples that tested positive per the total number of samples tested.

Direct qPCR amplification from pecan liquid endosperm as template was performed to determine the presence of *X. fastidiosa* in developing immature pecan seeds. Amplification of the expected *Xylella-*specific amplicon was improved upon dilution of the endosperm at 1:50, 1:75, and 1:100 (v/v). The proportion of liquid endosperm from developing seeds per the total number of seeds in which *X. fastidiosa* was detected was variable (Figure 1). *X. fastidiosa* was undetected in 4 out of 18 trees. The highest proportion of seeds testing positive for *X. fastidiosa* was 60% in cv. Wichita (Tree 6).

Quantitative PCR using isolated DNA as template was performed to detect the presence of *X. fastidiosa* in mature pecan seeds. A total of 72 seeds (10 seeds from TX1-NMU5 and 12 seeds from NMU6) were tested by qPCR. TX1-NMU6 seeds all tested positive for *X. fastidiosa*. The percentage of pecan seeds that were positive ranged from 50% (NMU1 and NMU5) to 90% (NMU 4; Table 1).

**Figure 2:**
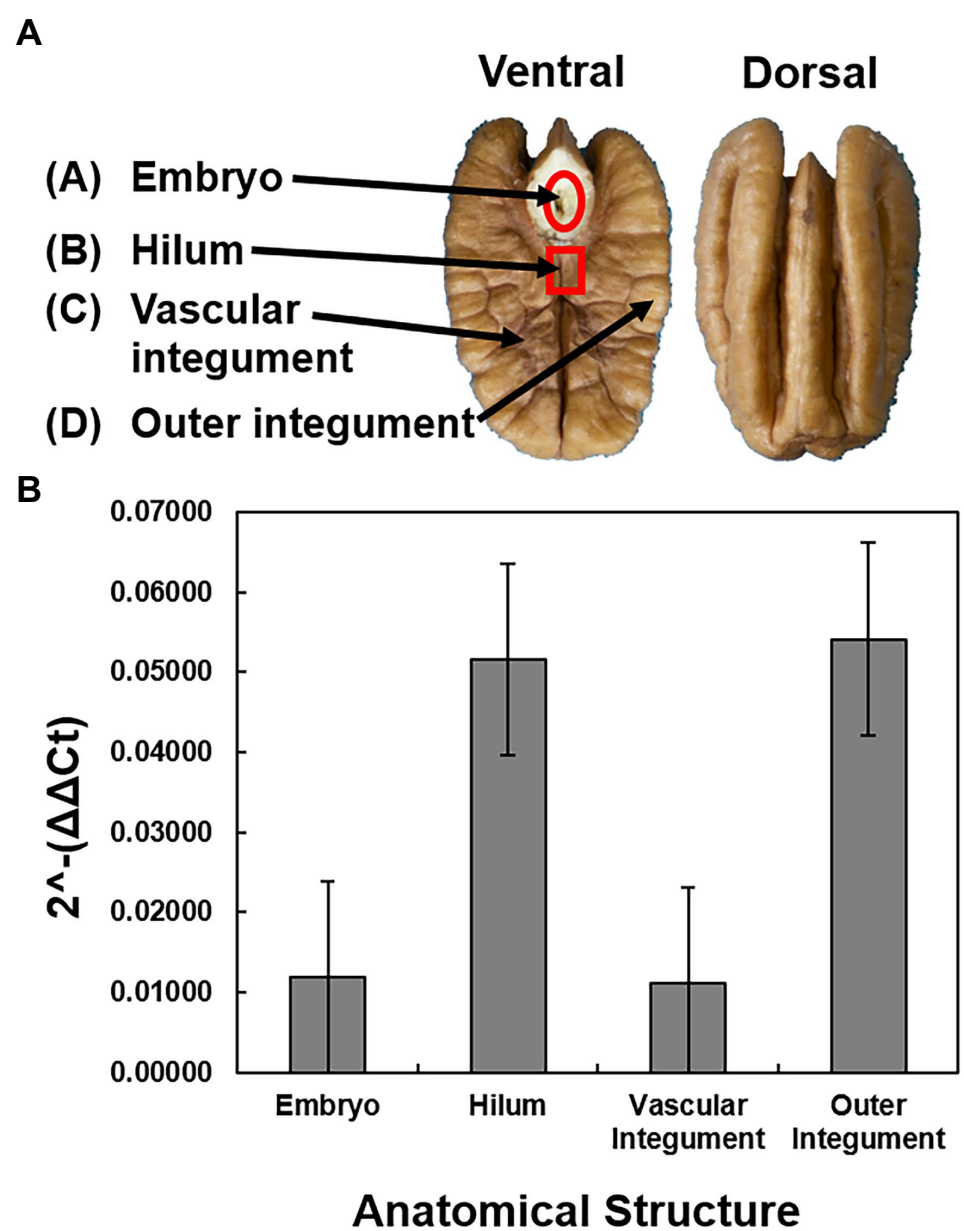
Sampling and quantification of *X. fastidiosa* in different anatomical structures of cultivar Cape Fear seeds. Four anatomical structures were dissected from four pecan seeds from a pecan bacterial leaf scorch diseased mother tree. **(A)** Anatomical diagram of a pecan seed. The pecan seed is cut across the longitudinal plane of the middle septum, with both the ventral and dorsal sections represented. Arrows indicate the embryo, hilum, vascular integument, and outer integument, respectively. **(B)** The 2^-ΔΔCt^ of *X. fastidiosa* DNA amplification normalized to pecan DNA amplification within each anatomical structure was estimated using qPCR. Error bars indicate standard errors of the mean values.

The *X. fastidiosa* DNA concentration was measured and compared in four anatomical parts of the seed to determine the pathogen localization patterns in the seed. The hilum and outer integument were found to have the highest concentrations of *X. fastidiosa* DNA when compared to the embryo and the vascular integument, but were not significantly different (Figure 2).

### Relationship Between *X. fastidiosa* Concentration and Pecan Seed Density to Germination and Growth Rate of Seedlings

**Figure 3:**
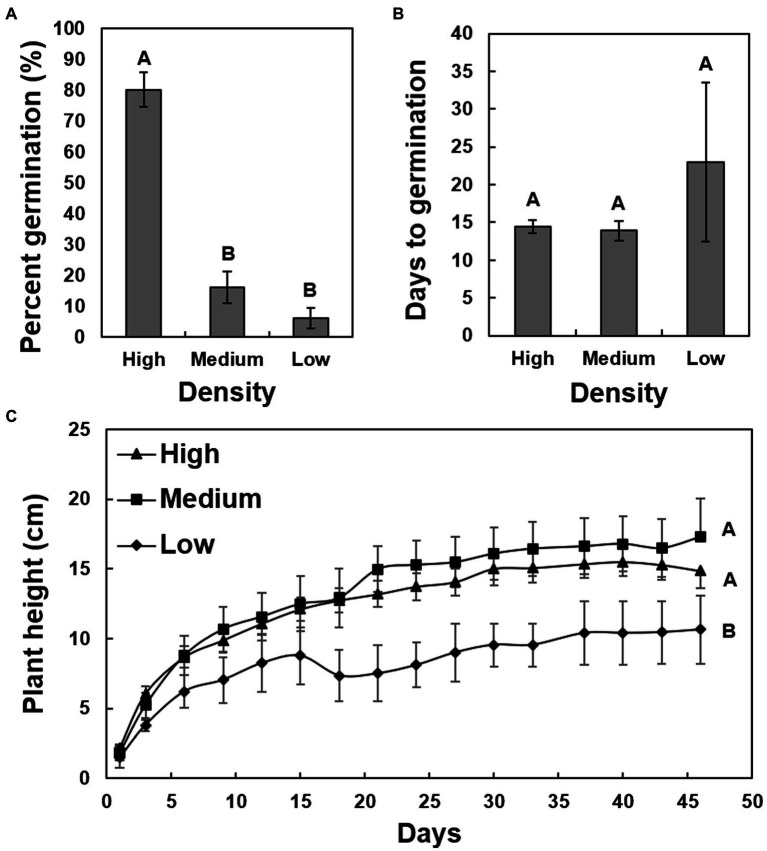
The impact of seed density on the germination and growth rate of seedlings of cultivar Cape Fear. A total of 150 seeds were collected from a pecan bacterial leaf scorch-diseased tree, measured for seed density, and separated into three groups. The average seed density of each group was as follows: high-density seed (0.72 g/cm^3^), medium-density seed (0.62 g/cm^3^), and low seed density (0.52 g/cm^3^). **(A)** Comparison of germination (%) of high-, medium-, and low-density seeds. **(B)** The average days to germination for high-, medium-, and low-density seeds. **(C)** Growth rate of seedlings germinated from the three different categories of seeds. Error bars indicated standard errors of the mean values. Means with different letters are significantly different based on Tukey’s HSD (*α* = 0.05).

**Figure 4:**
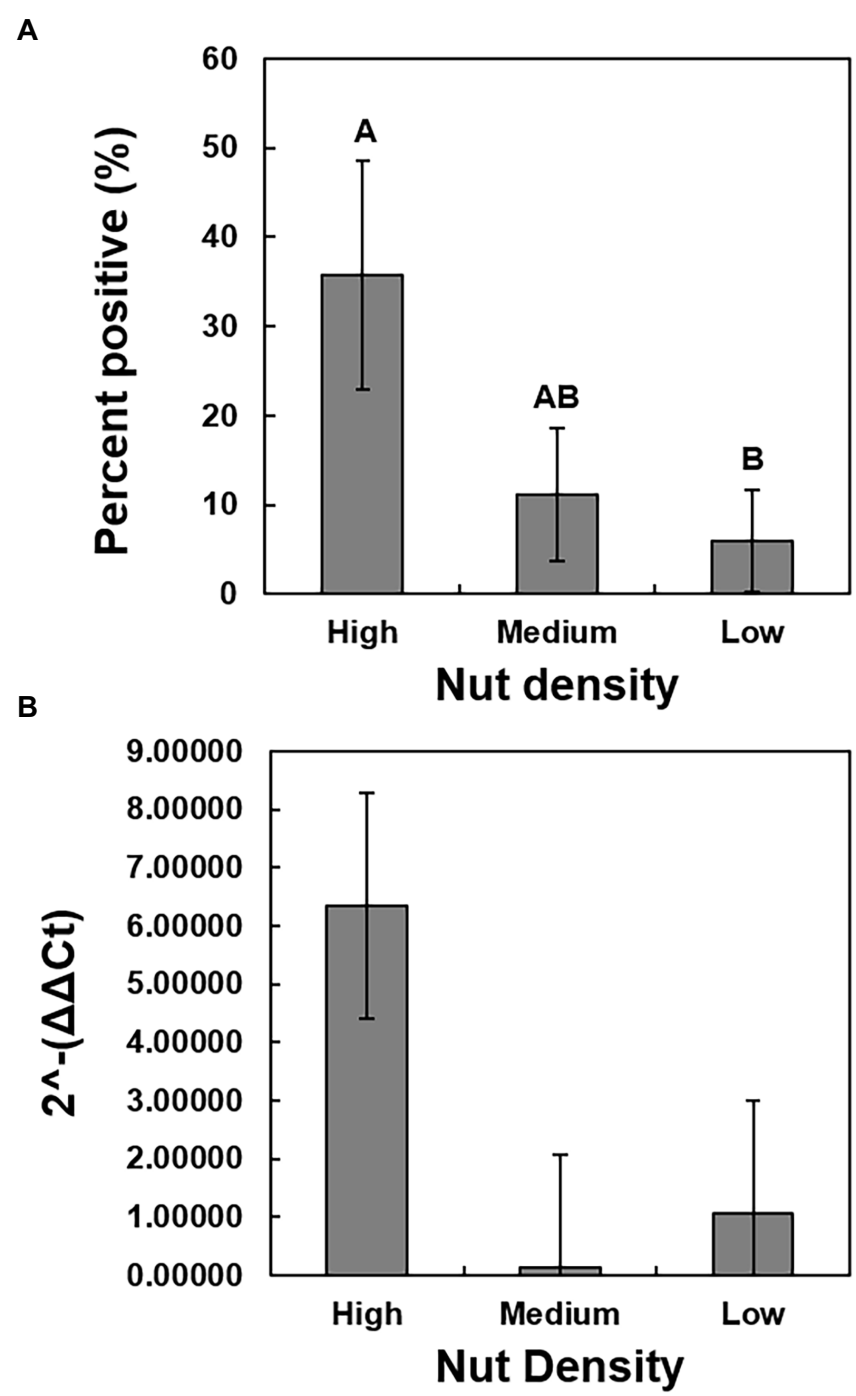
The relationship between seed density and the presence and concentration of *X. fastidiosa*. A total of 150 seeds were collected from pecan bacterial leaf scorch-diseased tree of cultivar Cape Fear and separated by density into three groups, based on seed density. The average seed density of each group was as follows: high-density seed (0.72 g/cm^3^), medium-density seed (0.62 g/cm^3^), and low seed density (0.52 g/cm^3^). Prior to planting, the seeds were bisected, and the section not containing the embryo was harvested and the gDNA isolated. qPCR analysis was performed on a subset of 25 seeds per group to determine the presence of *X. fastidiosa*. **(A)** The incidence (%) of seeds that tested positive for the presence of *X. fastidiosa*. **(B)** The 2^-ΔΔCt^ of *X. fastidiosa* DNA amplification normalized to pecan DNA amplification in the high-, medium-, and low-density seeds. Error bars indicated standard errors of the mean values. Means with different letters are significantly different based on Tukey’s HSD (*α* = 0.05).

A chi-square analysis of percent germination of low-, medium-, and high-density seeds revealed that germination was significantly higher in the high-density seeds (*χ*^2^ = 71.8360, *p* < 0.0001), whereas the medium- and low-density seeds had reduced germination of less than 20% and less than 10%, respectively (Figure 3A). The average days to germination were numerically slightly elevated in low-density seeds but were not significantly different when compared to the other two groups (Figure 3B). The overall average number of days to germination was approximately 15 days. The growth rate of seedlings originating from low-density seeds was significantly reduced (*F* = 13.92, *p* < 0.0001) in comparison to those germinating from the medium- and high-density seeds (Figure 3C). There was no significant difference between the growth rates of seedlings originating from the medium and high-density seeds. The high-density seed group had a significantly greater proportion of samples that tested positive for the presence of *X. fastidiosa* by qPCR than the low-density seed group (Figure 4A), but the *X. fastidiosa* DNA concentration was not significantly different among the seed density categories.

### Evidence of the Presence of *X. fastidiosa* in Seedlings

**Figure 5:**
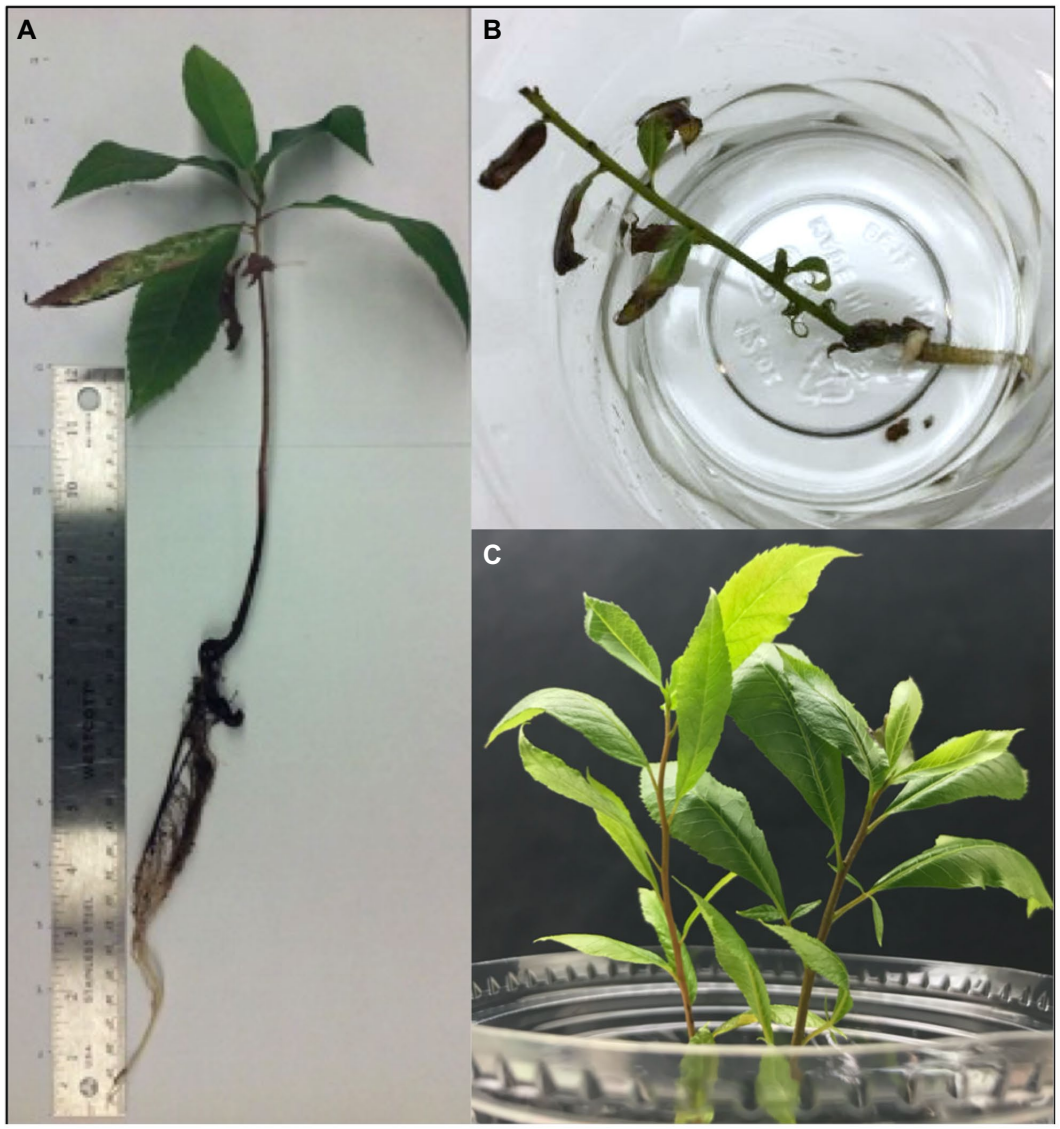
Symptoms of PBLS caused by *X. fastidiosa* in seedlings. **(A)** and **(B)** Seedlings grown from NM pecan seeds exhibiting PBLS symptoms. **(C)** Healthy pecan seedlings.

One hundred and fourteen seeds were planted into potting soil to determine the presence of *X. fastidiosa* in germinated pecan seedlings. Of those, only 32 seeds germinated and grew into seedlings (Table 1), and 2 of the seedlings died and thus, were not used for the study. Of the 30 remaining seedlings, nine exhibited classic PBLS symptoms that included tan to brown leaf tips and/or edges, necrosis along leaf margins extending toward the midrib of leaflets, and leaf abscission (Figure 5). qPCR confirmed the presence of *X. fastidiosa* in dissected seedling tissue and revealed a higher incidence of detection in the shoots followed by taproots, leaves including petioles, and fibrous roots, respectively (Table 2). Of the 30 seedlings tested, 27 tested positive for *X. fastidiosa* in at least one plant part tested (Table 1). It is important to note that 18 seedlings tested positive for the presence of *X. fastidiosa* but did not display PBLS symptoms.

**Table 2:** *Xylella fastidiosa* detection in seedling tissue.

Seedling tissue type	Incidence (% of samples) of detection for *X. fastidiosa*
Leaves/Petioles	47% (14/30)
Stems	60% (18/30)
Taproots	50% (15/30)
Fibrous roots	23% (7/30)

The incidence of tissues from seedlings tested that were positive for the presence of *X. fastidiosa* by PCR amplification and subsequent sequencing (using the NMU03 primer set) is indicated.

**Figure 6:**
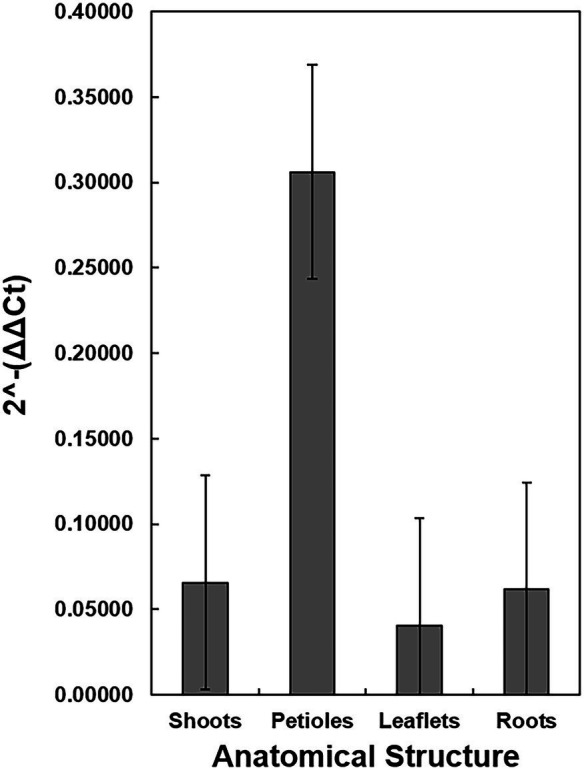
Detection and Quantification of *X. fastidiosa* DNA in different anatomical structures of c.v. Cape Fear seedlings. Eight seedlings (cultivar Cape Fear) were harvested and the four anatomical structures (petioles, apical shoot, tap root, and leaflets) were dissected using a scalpel. The 2^-ΔΔCt^ of *X. fastidiosa* DNA amplification normalized to pecan DNA amplification of each sample was estimated by qPCR. Error bars indicate standard errors of the mean values.

Additional dissections were made in eight TX1 seedlings to determine the relative quantity of *X. fastidiosa* DNA in the different anatomical parts of the plant. All of the TX1 seedlings had classic symptoms of PBLS, as previously described, by 3 months of growth. Comparisons of the quantity of *X. fastidiosa* in petioles, shoots, leaflets, and roots of TX1 seedlings (Figure 6), revealed that petioles had the highest concentrations of *X. fastidiosa* DNA as represented by 2^-ΔΔCt^. However, due to high variation, the differences in concentration were not significant among the different anatomical parts.

### Sequence Alignment and Phylogeny of *X. fastidiosa* From Pecan

**Figure 7:**
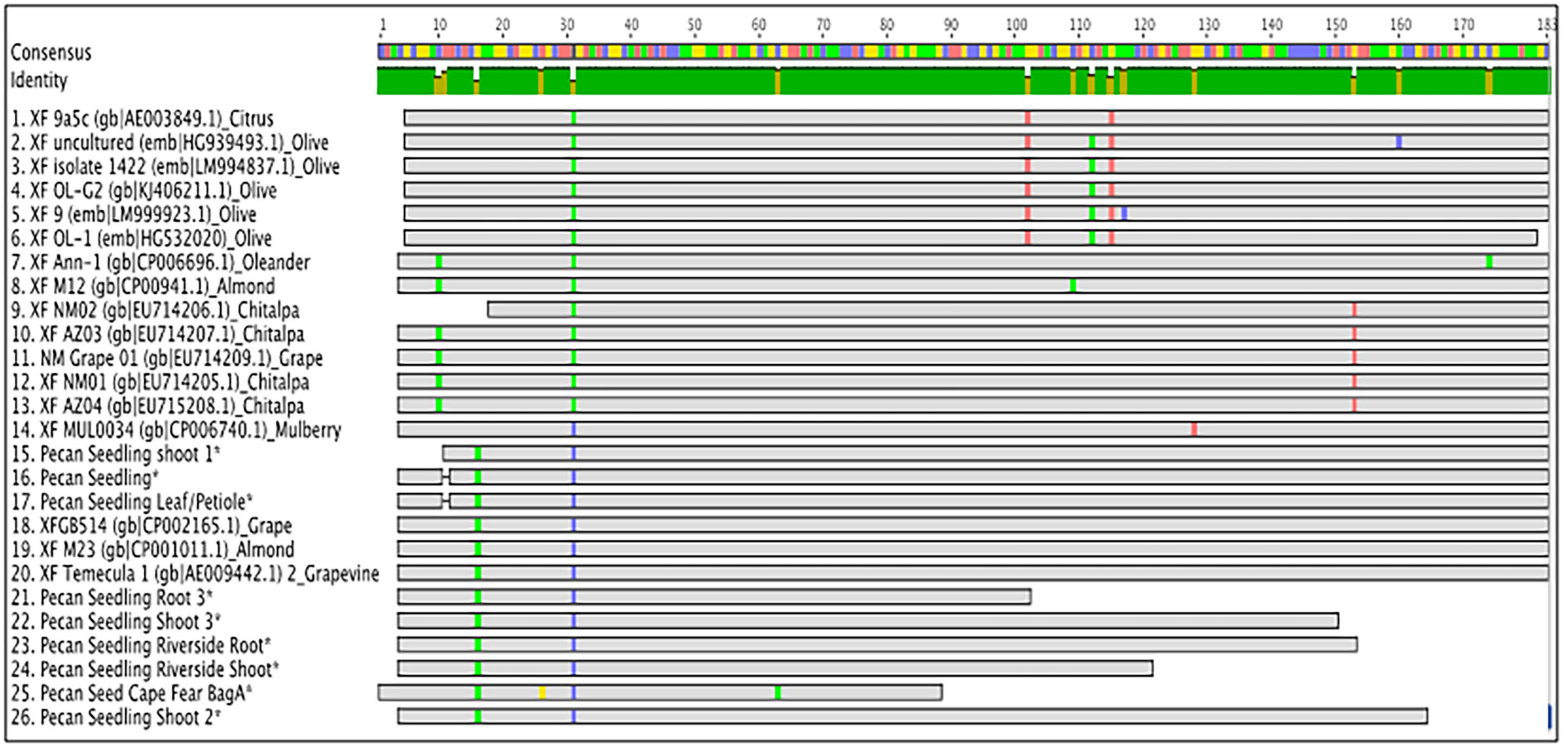
HL amplicon sequence alignment. Geneious Prime sequence alignment of HL amplicons from developing and mature pecan seeds and germinated seedlings compared to HL sequences deposited in GenBank (accession numbers indicated on alignment).

BLASTn [National Institute for Biotechnology Information (NCBI), Bethesda, MD] analysis of amplicon sequences confirmed a 98.96–100% identity to *X. fastidiosa*. The sequence alignment of the HL amplicons indicated that the sequences differed by a few base pairs (Figure 7). A nearest neighbor phylogenetic tree was constructed with HL sequences from this study along with and with *X. fastidiosa* HL sequences from GenBank (Supplementary Figure 1). The *X. fastidiosa* HL sequences from pecan tissues grouped with the *X. fastidiosa* HL sequences from several species including grape, chitalpa, almond, mulberry, and Oleander, but were distinct from HL sequences from olive and citrus (Supplementary Figure 1). Due to the conserved nature of the HL amplicon sequence, we are not able to determine which subspecies of *X. fastidiosa* was present in the pecan seeds and seedlings we sampled. The sequences reported in this study were submitted to BioProjects (PRJNA791935; PRJNA795489) in the NCBI database.

## Discussion

### *X. fastidiosa* Transmission From Seed to Seedling

We detected *X. fastidiosa* in underdeveloped fruit at the water stage, in fully developed seeds, and in germinated seedlings using qPCR and sequencing. Several possible modes of transmission of *X. fastidiosa* have been considered previously, including moderate to high transmission rates by xylem-feeding insects and transmission by vegetative propagation through the grafting of pecan rootstocks and scions (Sanderlin and Melanson, 2006, 2008, 2010). The detection of *X. fastidiosa* in the liquid endosperm of pecan seeds has been demonstrated previously by PCR and ELISA in at least three different cultivars (Hilton et al., 2017, 2020), and we not only confirmed these observations but also demonstrated vertical transmission to seedlings germinating from *Xylella-*contaminated seeds that were harvested from PBLS symptomatic mother pecan trees.

Pecan seed development can be separated into three stages, histodifferentiation, maturation, and post-abscission (Jeyaretnam et al., 1999). During histodifferentiation and early maturation phases of the seed, water content dramatically increases as it travels through the vascular bundles to the inner tissue of the funiculus, which meets near the base of the integument before branching out into a complex system throughout the kernel seed coat (Woodroof and Chapman-Woodroof, 1927; Jeyaretnam et al., 1999). The hilum is an elliptical scar that marks the site of previous attachment to the funiculus, and in which we found some of the highest concentrations of *X. fastidiosa* DNA in the pecan seeds tested. As was the case in sweet orange, *X. fastidiosa* was also reported to be present in the cotyledons and kernel seed coat representing the vascular and outer integument, as well as the embryo (Coletta-Filho et al., 2014). Further research is needed to understand the movement of *X. fastidiosa* through the peduncle xylem tissue and into the vascular system of developing seeds, particularly in the presence of PBLS symptoms on shoots bearing fruit clusters.

*Xylella fastidiosa* was detected in the pecan seedling progeny originating from maternal trees bearing seeds found to contain the bacterium. This is in contrast to the report of Coletta-Filho et al. (2014), in which *X. fastidiosa* was not detected in the seedling progeny from infected sweet orange seeds. Eight pecan seedlings obtained from the TX1 mother tree that germinated from high-density seeds were harvested after 3 months and dissected into four parts. High DNA concentrations of the bacterium were found in petioles, but DNA of *X. fastidiosa* was also detected in shoots, roots, and leaflets. This suggests similar patterns of localization of *X. fastidiosa* as within other host crop species, in which the bacterium translocates through the vascular system and predominantly colonizes petioles (Fry and Milholland, 1990; Almeida et al., 2001; Sanderlin and Heyderich-Alger, 2003). It has been proposed that while in the host plant, *X. fastidiosa* assumes a translocation phase that can be characterized by extensive vessel colonization at low cell titers (Chatterjee et al., 2008). The potential for low titers of the *X. fastidiosa* bacterium traveling from seeds into meristematic tissue emphasizes the need for established sampling protocols when diagnosing the pathogen in seedlings to ensure disease-free rootstocks and to minimize the risk of false negatives.

### Association Between *X. fastidiosa* Colonization and Seed Density

Surprisingly, a significantly greater proportion of high-density seeds tested positive for the presence of *X. fastidiosa* by qPCR compared to low-density seeds. *X. fastidiosa* DNA concentration was also elevated in high-density seeds, providing evidence that the bacterium can colonize seeds without negatively influencing development or overall density. The fact that infection is not associated with seed density eliminates the possibility for seed culling as a method of reducing *Xylella-*contaminated seeds that might be of low density, an effective approach for limiting disease in other food crops (Morrison, 1999; Gelin et al., 2006; Miller et al., 2013). In a study of seed transmission of *X. fastidiosa* subsp. *pauca* in sweet orange, embryo weight and germination of CVC-infected seed was reduced by 25 and 9%, respectively (Coletta-Filho et al., 2014). The results were inconsistent with our findings, as despite increased concentrations *X. fastidiosa* DNA, high-density seed germination was significantly greater when compared to medium- and low-density seeds, and the growth rates of germinated seedlings in the first 3 months were unaffected by infection status. The long-term impact of *X. fastidiosa* in these seedlings needs to be further evaluated.

The mechanisms for the development of disease symptoms in *Xylella-*infected hosts have not been fully elucidated. However, the scorch symptoms that are observed have been attributed to the occlusion of xylem vessels due to the presence of *X. fastidiosa* biofilm that negatively impact water transpiration (Hopkins, 1981; Newman et al., 2003). Newman et al. (2003) proposed that the aggregation of nonviable bacterial cells during the formation of a biofilm is a byproduct of the obstruction to intercellular mobility resulting in disease symptoms. Thus, in our study, greater *X. fastidiosa* titers in high-density seeds may be explained if vascular translocation of the bacterium was achieved due to the lack of an obstructive biofilm in the xylem. A recent study utilized a *X. fastidiosa-*specific fluorescence *in situ* hybridization (FISH) probe to microscopically monitor the localization patterns of the OQDS pathogen in the xylem tissue of olive petioles and branches (Cardinale et al., 2018). It would be useful to replicate this technique of FISH-Confocal laser scanning microscopy (CLSM) analysis in a future study to determine how *X. fastidiosa* localizes and ultimately, translocates, through seed and seedling pecan tissues.

### Source of Infection in Seed

The mechanism of seed infection is unknown since there are several possible modes of transmission by *X. fastidiosa*. There is evidence for seed-to-seedling transmission of *Xanthomonas* spp. [*Xylella* and *Xanthomonas* are closely related genera, both belonging to the family Xanthomonadaceae (Wells et al., 1987)]. A study by Brinkerhoff and Hunter (1963) found *Xanthomonas malvacearum* to be present in the embryos and seed coats of cotton. *Xanthomonas campestris* was also found to be efficiently transmitted from seed to seedling from inoculated bean flowers (Darrasse et al., 2010). Zaumeyer (1930) traced the passage of *Xanthomonas axonopodis* pv. *phaseoli* from bean plants to pods and found that the bacterium in the xylem vessels of the stem travels through the vascular system by way of the raphe and into the seed coat. Once in the seed coat, *Xanthomonas* travels through the intercellular spaces and spreads throughout the tissue of the seed (Zaumeyer, 1930). In pecan seed development, the cotyledons have folds with a thin membrane containing vascular bundles (Woodroof and Chapman-Woodroof, 1927). *X. fastidiosa* can move across and/or through xylem elements presenting possible direct maternal transmission (Purcell and Finlay, 1979) as a first mechanism of seed infection.

Another possible source of infection is through xylem-feeding insects. Insects are known to vector *X. fastidiosa* and could be feeding on the xylem vessels of the pecan shuck. For example, pecan spittlebugs have been documented to congregate in colonies on terminals, peduncles, and nutlets in the tree canopy during the early period of the growing season (Harris, 1983). Extensive vector feeding in the proximity of and on developing fruit may provide an opportunity for *Xylella* to enter the seed through xylem-feeding insects, including pecan spittlebugs, or other known vectors of the bacterium (Sanderlin and Melanson, 2010). As the bacterium is introduced into the xylem vessels of the shuck, the bacterium could travel through the xylem and into the seed.

It is important to point out that we were not able to determine the subspecies of *X. fastidiosa* present in the seeds and resulting seedlings of our study. As pecan and *X. fastidiosa* both originated in the “New World,” it is possible that the natural microbiome for pecan includes characteristic *X. fastidiosa* subspecies (Thompson and Grauke, 1991; Nunney et al., 2013; Randall et al., 2021). In previous studies, *X. fastidiosa* subsp. *multiplex* has been shown to cause disease in pecans (Melanson et al., 2012; Sanderlin, 2017). As *X. fastidiosa* is reportedly endemic in pecan in southern pecan growing regions in the United States, it is likely that many pecan trees may be inhabited by the bacterium (Sanderlin and Heyderich-Alger, 2000; Hilton et al., 2017; Bock et al., 2018). Although beyond the scope of this study, it is possible that avirulent *X. fastidiosa* subspecies naturally inhabit pecan with no consequence of disease symptomology, at least with the known exception of disease-causing strains of *X. fastidiosa* subsp. *multiplex*.

Pecan seedlings of various cultivars are the standard rootstock source for pecan nurseries (Thompson and Conner, 2012). Thus, the potential for seed transmission of *Xylella* in pecan is a threat to nursery production of pecan trees if the seed of infected trees is used as a rootstock source. The results of our study showed that out of 27 seedlings that were positive for *X. fastidiosa* only nine exhibited PBLS symptoms. Knowledge of *Xylella* in pecan is limited to only a handful of studies. Understanding the influence of *X. fastidiosa* on pecan seed development and viability is critical for assessing the disease risk to the industry nationally and internationally and to the natural environment. This study provides preliminary evidence to support the likelihood of seed-to-seedling transmission in pecan.

Due to the burgeoning global movement of plant material, major challenges include disease surveillance, detection, and diagnosis (Jeger et al., 2021). Understanding alternative modes of pathogen dissemination are critical to gauging risks and developing mitigation and phytosanitary protocols and regulation. Characterizing the role of seed transmission of *X. fastidiosa* may change perspectives in other seed-producing crops besides pecan and help reduce the risk of pathogen spread within areas or to new regions.

## Data Availability Statement

The datasets presented in this study can be found in online repositories. The names of the repository/repositories and accession number(s) can be found in the article/Supplementary Material.

## Author Contributions

KC and AH contributed equally to this manuscript as co-first authors and wrote the draft manuscript. KC, AH, RS, CB, RH, LG, and JR conceived and designed the study. KC, AH, RS, and JR performed the experiments. RH, XW, and LG contributed to logistics and material collection. JR, LG, XW, and Y-KJ contributed working space, materials, and equipment and provided funding for the research. All authors participated in the interpretation of the results and read and approved the manuscript.

## Funding

This work was supported by USDA-ARS CRIS 3091-21000-042-00D “Management of the National Collection of Carya Genetic Resources and Associated Information”; USDA-ARS CRIS 6042-21220-012-00-D “Mitigating Alternate Bearing of Pecan”; National Plant Germplasm System Grant 58-3091-6-022 “Screening *X. fastidiosa* in the USDA ARS National Collection of Genetic Resources for Carya”; USDA NIFA SCRI-2016-51181-25408 and the Southern Integrative Pest Management (IPM) Center Program (project #1702922). Graduate study was supported by a research support agreement (58-3091-6-030) from the USDA-ARS Southern Plains Agricultural Research Center in College Station, TX.

## Conflict of Interest

The authors declare that the research was conducted in the absence of any commercial or financial relationships that could be construed as a potential conflict of interest.

## Publisher’s Note

All claims expressed in this article are solely those of the authors and do not necessarily represent those of their affiliated organizations, or those of the publisher, the editors and the reviewers. Any product that may be evaluated in this article, or claim that may be made by its manufacturer, is not guaranteed or endorsed by the publisher.

## Footnotes

^1^ https://www.geneious.com

^2^ https://www.ncbi.nlm.nih.gov/genbank/

## References

[ref1] Almeida R. Pereira E. Purcell A. Lopes J. (2001). Multiplication and movement of a citrus strain of *Xylella fastidiosa* within sweet orange. Plant Dis. 85, 382–386. doi: 10.1094/PDIS.2001.85.4.382, PMID: 30831970

[ref4] Bock C. H. Oliver J. E. Chen C. Hotchkiss M. H. Stevenson K. L. Wang X.. (2018). Pecan bacterial leaf scorch, caused by *Xylella fastidiosa*, is endemic in Georgia pecan orchards. Plant Health Prog. 19, 284–287. doi: 10.1094/PHP-08-18-0045-S

[ref5] Brinkerhoff L. Hunter R. (1963). Internally infected seed as a source of inoculum for the primary cycle of bacterial blight of cotton. Phytopathology 53, 1397–1401.

[ref6] Cardinale M. Luvisi A. Meyer J. B. Sabella E. De Bellis L. Cruz A. C.. (2018). Specific fluorescence in situ hybridization (FISH) test to highlight colonization of xylem vessels by *Xylella fastidiosa* in naturally infected olive trees (*Olea europaea* L.). Frontiers. Plant Sci. 9:431. doi: 10.3389/fpls.2018.00431, PMID: 29681910 PMC5897508

[ref7] Chatterjee S. Almeida R. P. Lindow S. (2008). Living in two worlds: the plant and insect lifestyles of *Xylella fastidiosa*. Annu. Rev. Phytopathol. 46, 243–271. doi: 10.1146/annurev.phyto.45.062806.094342, PMID: 18422428

[ref8] Coletta-Filho H. D. Carvalho S. A. Silva L. F. C. Machado M. A. (2014). Seven years of negative detection results confirm that *Xylella fastidiosa*, the causal agent of CVC, is not transmitted from seeds to seedlings. Eur. J. Plant Pathol. 139, 593–596. doi: 10.1007/s10658-014-0415-8

[ref01] Dandekar A. M. Jacobson A. Ibáñez A. M. Gouran H. Dolan D. L. Agüero C. B.. (2019). Trans-graft protection against Pierce’s disease mediated by transgenic grapevine rootstocks. Frontiers in plant science 10, 84 30787937 10.3389/fpls.2019.00084 PMC6372540

[ref9] Darrasse A. Darsonval A. Boureau T. Brisset M.-N. Durand K. Jacques M. A.. (2010). Transmission of plant-pathogenic bacteria by nonhost seeds without induction of an associated defense reaction at emergence. Appl. Environ. Microbiol. 76, 6787–6796. doi: 10.1128/AEM.01098-10, PMID: 20729326 PMC2953029

[ref10] EFSA (European Food Safety Authority) (2020). Scientific report on the update of the *Xylella* spp. host plant database – systematic literature search up to 30 June 2019. EFSA J. 18, 6114–6178. doi: 10.2903/j.efsa.2020.6114, PMID: 32874307 PMC7448098

[ref11] Ellis E. A. McEachern G. R. Clark S. Cobb B. G. (2010). Ultrastructure of pit membrane dissolution and movement of *Xylella fastidiosa* through pit membranes in petioles of *Vitis vinifera*. Botany 88, 596–600. doi: 10.1139/B10-025

[ref02] Francis M. Lin H. Rosa J. C. L. Doddapaneni H. Civerolo E. L. (2006). Genome-based PCR primers for specific and sensitive detection and quantification of *Xylella fastidiosa*. Eur. J. Plant Pathol. 115, 203–213.

[ref12] Fry S. Milholland R. (1990). Multiplication and translocation of *Xylella fastidiosa* in petioles and stems of grapevine resistant, tolerant, and susceptible to Pierce’s disease. Phytopathology 80, 61–65. doi: 10.1094/Phyto-80-61

[ref13] Gelin J. Arelli P. Rojas-Cifuentes G. (2006). Using independent culling to screen plant introductions for combined resistance to soybean cyst nematode and sudden death syndrome. Crop Sci. 46, 2081–2083. doi: 10.2135/cropsci2005.12.0505

[ref14] Grauke L. Mendoza-Herrera M. A. Miller A. J. Wood B. W. (2011). Geographic patterns of genetic variation in native pecans. Tree Genet. Genom. 7, 917–932. doi: 10.1007/s11295-011-0384-4

[ref15] Grauke L. J. Thompson T. E. (1995). Patterns of rootstock usage in the pecan industry. HortScience 30, 431f–431f. doi: 10.21273/HORTSCI.30.3.431f

[ref16] Grauke L. J. Wood B. W. Harris M. K. (2016). Crop vulnerability: Carya. HortScience 51, 653–663. doi: 10.21273/HORTSCI.51.6.653

[ref17] Harris M. K. (1983). Integrated pest management of pecans. Annu. Rev. Entomol. 28, 291–318. doi: 10.1146/annurev.en.28.010183.001451, PMID: 35066591

[ref18] Hartung J. Nian S. Lopes S. Ayres A. Brlansky R. (2014). Lack of evidence for transmission of *Xylella fastidiosa* from infected sweet orange seed. J. Plant Pathol. 96, 497–506.

[ref19] Hilton A. Jeong M. Hsu J. H. Cao F. Choi W. Wang X.. (2021). Thermal treatment using microwave irradiation for the phytosanitation of *Xylella fastidiosa* in pecan graftwood. PLoS One 16:e0244758. doi: 10.1371/journal.pone.0244758, PMID: 33471831 PMC7816998

[ref20] Hilton A. Jo Y.-K. Cervantes K. Stamler R. French J. M. Heerema R. J.. (2017). First report of pecan bacterial leaf scorch caused by *Xylella fastidiosa* in pecan (*Carya illinoinensis*) in Arizona, New Mexico, California, and Texas. Plant Dis. 101:1949. doi: 10.1094/PDIS-02-17-0298-PDN

[ref21] Hilton A. Wang X. Zhang M. Cervantes K. French J. Randall J. J.. (2020). Improved methods for detecting *Xylella fastidiosa* in pecan and related *Carya* species. Eur. J. Plant Pathol. 157, 899–918. doi: 10.1007/s10658-020-02050-5

[ref22] Hopkins D. (1981). Seasonal concentration of the Pierce’s disease bacterium in grapevine stems, petioles, and leaf veins. Phytopathology 71, 415–418. doi: 10.1094/Phyto-71-415

[ref23] Jeger M. Beresford R. Bock C. Brown N. Fox A. Newton A.. (2021). Global challenges facing plant pathology: multidisciplinary approaches to meet the food security and environmental challenges in the mid-twenty-first century. CABI Agri. Biosci. 2, 1–18. doi: 10.1186/s43170-021-00042-x

[ref24] Jeyaretnam B. Levi A. Phatak S. C. Wetzstein H. Y. (1999). Changes in growth, water content and protein reflect embryo development in pecan (*Carya illinoinensis*). J. Hortic. Sci. Biotechnol. 74, 315–320. doi: 10.1080/14620316.1999.11511115

[ref04] Kearse M. Moir R. Wilson A. Stones-Havas S. Cheung M. Sturrock S.. (2012). Geneious Basic: an integrated and extendable desktop software platform for the organization and analysis of sequence data. Bioinformatics 28, 1647–1649. 22543367 10.1093/bioinformatics/bts199 PMC3371832

[ref25] Lazarotto M. Milanesi P. Muniz M. Reiniger L. Beltrame R. Harakava R.. (2014). Morphological and molecular characterization of *Fusarium* spp pathogenic to pecan tree in Brazil. Genet. Mol. Res. 13, 9390–9402. doi: 10.4238/2014.November.11.5, PMID: 25501150

[ref26] Li W. B. Pria W. D. Jr. Lacava P. M. Qin X. Hartung J. S. (2003). Presence of *Xylella fastidiosa* in sweet orange fruit and seeds and its transmission to seedlings. Phytopathology 93, 953–958. doi: 10.1094/PHYTO.2003.93.8.953, PMID: 18943861

[ref27] Martelli G. P. Boscia D. Porcelli F. Saponari M. (2016). The olive quick decline syndrome in south-East Italy: a threatening phytosanitary emergency. Eur. J. Plant Pathol. 144, 235–243. doi: 10.1007/s10658-015-0784-7

[ref28] Marzolo G. (2015). Pecans. Agric. Market. Res. Cent. Available at: http://www.agmrc.org/commodities-products/nuts/pecans

[ref29] Melanson R. Sanderlin R. McTaggart A. Ham J. (2012). A systematic study reveals that *Xylella fastidiosa* strains from pecan are part of *X. fastidiosa* subsp. *multiplex*. Plant Dis. 96, 1123–1134. doi: 10.1094/PDIS-09-11-0730-RE, PMID: 30727050

[ref30] Miller S.A. Lewis Ivey M.L. Baysal-Gurel F. Xiulan X. (2013). “A Systems Approach to Tomato Disease Management”: International Society for Horticultural Science (ISHS), Leuven, Belgium), 167–172.

[ref31] Mollenhauer H. H. Hopkins D. L. (1974). Ultrastructural study of Pierce’s disease bacterium in grape xylem tissue. J. Bacteriol. 119, 612–618. doi: 10.1128/jb.119.2.612-618.1974, PMID: 4855307 PMC245648

[ref32] Morelli M. García-Madero J. M. Jos Á. Saldarelli P. Dongiovanni C. Kovacova M.. (2021). *Xylella fastidiosa* in olive: A review of control attempts and current management. Microorganisms 9, 1771–1791. doi: 10.3390/microorganisms9081771, PMID: 34442850 PMC8397937

[ref33] Morrison R. H. (1999). Sampling in seed health testing. Phytopathology 89, 1084–1087. doi: 10.1094/PHYTO.1999.89.11.1084, PMID: 18944666

[ref03] Nascimento R. Gouran H. Chakraborty S. Gillespie H. W. Almeida-Souza H. O. Tu A.. (2016). The type II secreted lipase/esterase LesA is a key virulence factor required for *Xylella fastidiosa* pathogenesis in grapevines. Scientific Reports 6, 1–17. 26753904 10.1038/srep18598 PMC4709584

[ref34] Newman K. L. Almeida R. P. Purcell A. H. Lindow S. E. (2003). Use of a green fluorescent strain for analysis of *Xylella fastidiosa* colonization of *Vitis vinifera*. Appl. Environ. Microbiol. 69, 7319–7327. doi: 10.1128/AEM.69.12.7319-7327.2003, PMID: 14660381 PMC310014

[ref35] Nunney L. Vickerman D. B. Bromley R. E. Russell S. A. Hartman J. R. Morano L. D.. (2013). Recent evolutionary radiation and host plant specialization in the *Xylella fastidiosa* subspecies native to the United States. Appl. Environ. Microbiol. 79, 2189–2200. doi: 10.1128/aem.03208-12, PMID: 23354698 PMC3623259

[ref37] Oliver J. Cobine P. De La Fuente L. (2015). *Xylella fastidiosa* isolates from both subsp. *multiplex* and *fastidiosa* cause disease on southern highbush blueberry (*Vaccinium* sp.) under greenhouse conditions. Phytopathology 105, 855–862. doi: 10.1094/PHYTO-11-14-0322-FI, PMID: 25738552

[ref38] Paterson A. H. Brubaker C. L. Wendel J. F. (1993). A rapid method for extraction of cotton (*Gossypium* spp.) genomic DNA suitable for RFLP or PCR analysis. Plant Mol. Biol. Report. 11, 122–127. doi: 10.1007/BF02670470, PMID: 35193115

[ref39] Purcell A. H. Finlay J. (1979). Evidence for noncirculative transmission of Pierce’s disease bacterium by sharpshooter leafhoppers. Phytopathology 69, 393–395. doi: 10.1094/Phyto-69-393

[ref40] Randall J. J. Cervantes K. Ray D. K. Sanchez A. Mason K. Fisk J. N.. (2021). Insights into the impact of geography and genetics on the microbiome of *Carya illinoinensis*. Acta Hortic. 1318, 235–240. doi: 10.17660/ActaHortic.2021.1318.34

[ref41] Randall J. Rascon A. Heerema R. Potter M. (2015). Molecular mechanisms of pecan flower induction. Acta Hortic. 1070, 89–99. doi: 10.17660/ActaHortic.2015.1070.10

[ref42] Roper M. C. Greve L. C. Labavitch J. M. Kirkpatrick B. C. (2007). Detection and visualization of an exopolysaccharide produced by *Xylella fastidiosa* in vitro and in planta. Appl. Environ. Microbiol. 73, 7252–7258. doi: 10.1128/AEM.00895-07, PMID: 17827325 PMC2168192

[ref43] Rüter B. Hamrick J. Wood B. (1999). Genetic diversity within provenance and cultivar germplasm collections versus natural populations of pecan (*Carya illinoinensis*). J. Hered. 90, 521–528. doi: 10.1093/jhered/90.5.521

[ref44] Sanderlin R. (2005). Cultivar and seedling susceptibility to pecan bacterial leaf scorch caused by *Xylella fastidiosa* and graft transmission of the pathogen. Plant Dis. 89, 446–449. doi: 10.1094/PD-89-0446, PMID: 30795419

[ref45] Sanderlin R. (2017). Host specificity of pecan strains of *Xylella fastidiosa* subsp. *multiplex*. Plant Dis. 101, 744–750. doi: 10.1094/PDIS-07-16-1005-RE, PMID: 30678562

[ref46] Sanderlin R. Heyderich-Alger K. (2000). Evidence that *Xylella fastidiosa* can cause leaf scorch disease of pecan. Plant Dis. 84, 1282–1286. doi: 10.1094/PDIS.2000.84.12.1282, PMID: 30831868

[ref47] Sanderlin R. Heyderich-Alger K. (2003). Effects of pecan bacterial leaf scorch on growth and yield components of cultivar Cape Fear. Plant Dis. 87, 259–262. doi: 10.1094/PDIS.2003.87.3.259, PMID: 30812757

[ref48] Sanderlin R. Melanson R. (2006). Transmission of *Xylella fastidiosa* through pecan rootstock. HortScience 41, 1455–1456. doi: 10.21273/HORTSCI.41.6.1455, PMID: 30769528

[ref49] Sanderlin R. Melanson R. (2008). Reduction of *Xylella fastidiosa* transmission through pecan scion wood by hot-water treatment. Plant Dis. 92, 1124–1126. doi: 10.1094/PDIS-92-7-1124, PMID: 30769528

[ref50] Sanderlin R. Melanson R. (2010). Insect transmission of *Xylella fastidiosa* to pecan. Plant Dis. 94, 465–470. doi: 10.1094/PDIS-94-4-0465, PMID: 30754516

[ref51] Schmittgen T. D. Livak K. J. (2008). Analyzing real-time PCR data by the comparative C(T) method. Nat. Protoc. 3, 1101–1108. doi: 10.1038/nprot.2008.73, PMID: 18546601

[ref53] Schrader C. Schielke A. Ellerbroek L. Johne R. (2012). PCR inhibitors – occurrence, properties and removal. J. Appl. Microbiol. 113, 1014–1026. doi: 10.1111/j.1365-2672.2012.05384.x, PMID: 22747964

[ref56] Thompson T. E. Conner P. J. (2012). “Pecan,” in Fruit Breeding. eds. Badenes M. L. Byrne D. H. (New York: Springer), 771–801.

[ref57] Thompson T. E. Grauke L. J. (1991). Pecans and other hickories (*Carya*). Genet. Res. Temp. Fruit Nut Crop. 290, 839–906. doi: 10.17660/ActaHortic.1991.290.19

[ref58] USDA, N.A.S.S (2020). Pecan production.

[ref59] Vitanza S. (2015). Newsletter about integrated pest management for the El Paso valley. Issues Agric. 40, 1–4.

[ref60] Wakeling L. T. Mason R. L. D’Arc B. R. Caffin N. A. (2001). Composition of pecan cultivars Wichita and western Schley [*Carya illinoinensis* (Wangenh.) K. Koch] grown in Australia. J. Agric. Food Chem. 49, 1277–1281. doi: 10.1021/jf000797d, PMID: 11312850

[ref61] Wells J. M. Raju B. C. Hung H.-Y. Weisburg W. G. Mandelco-Paul L. Brenner D. J. (1987). *Xylella fastidiosa* gen. Nov., sp. nov: gram-negative, xylem-limited, fastidious plant bacteria related to *Xanthomonas spp*. Int. J. Syst. Evol. Microbiol. 37, 136–143.

[ref62] Wood B. W. Payne J. A. Grauke L. J. (1990). The rise of the US pecan industry. HortScience 25, 594–723. doi: 10.21273/HORTSCI.25.6.594

[ref63] Woodroof J. G. Chapman-Woodroof N. (1927). Pecan from flower to maturity. J. Agric. Res. 34, 1049–1065.

[ref64] Worley R. E. (1994). “Pecan production,” in Pecan Technology. ed. Santerre C. R. (Dordrecht: Springer), 12–38.

[ref65] Zaumeyer W.J. (1930). The Bacterial Blight of Beans Caused by Bacterium Phaseoli. Washington, DC: US Dept. of Agriculture.

[ref66] Zhang R. Peng F. Li Y. (2015). Pecan production in China. Sci. Hortic. 197, 719–727. doi: 10.1016/j.scienta.2015.10.035, PMID: 34784360

