# Supplementary figures and images for: Evidence for Seed Transmission of Xylella fastidiosa in Pecan (Carya illinoinensis)
Source: Front Plant Sci. 2022 Apr 8;13:780335. doi: 10.3389/fpls.2022.780335 (PMC9024359; doi:10.3389/fpls.2022.780335)

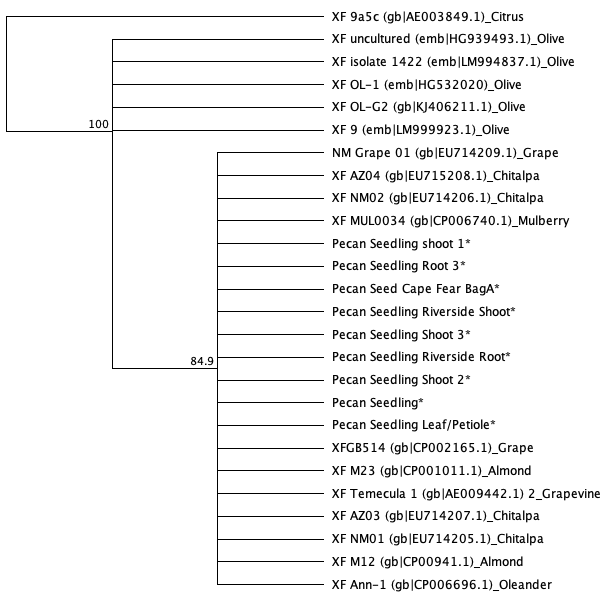

Supplement: Supplementary file 2 [file Image_1.PNG]
